# Evaluation of practical experiences of German speaking radiation oncologists in combining radiation therapy with checkpoint blockade

**DOI:** 10.1038/s41598-021-86863-2

**Published:** 2021-04-07

**Authors:** Kim M. Kraus, Julius C. Fischer, Kai J. Borm, Marco M. E. Vogel, Steffi. U. Pigorsch, Michal Devečka, Stephanie E. Combs

**Affiliations:** 1 Department of Radiation Oncology, School of Medicine, Technical University of Munich (TUM), Klinikum rechts der Isar, Munich, Germany; 2 Department of Radiation Sciences (DRS), Helmholtz Zentrum München (HMGU), Institute of Radiation Medicine (IRM), Neuherberg, Munich, Germany; 3 Deutsches Konsortium für Translationale Krebsforschung (DKTK), Partner Site Munich, Munich, Germany

**Keywords:** Oncology, Tumour immunology

## Abstract

The results of this survey reveal current clinical practice in the handling of combined radioimmunotherapy with Immune Checkpoint Inhibitors (RT + ICI). We aim to provide a basis to open a discussion for clinical application of RT + ICI by analyzation of experts’ assessment. We conducted a survey with 24 items with a focus on side effects of RT + ICI, common practice of scheduling and handling of adverse events. After pilot testing by radiation oncology experts the link to the online survey was sent to all members of the German Society of Radiation Oncology (DEGRO). In total, 51 radiation oncologists completed the questionnaire. Pulmonary toxicity under RT + ICI with ICIs was reported most frequently. Consensus was observed for bone and soft tissue RT of the limbs in favor for no interruption of ICIs. For cranial RT half of the participants do not suspend ICIs during normofractionated radiotherapy (nfRT) or stereotactic hypofractionated RT (SRT). More participants pause ICIs for central than for peripheral thoracic region. Maintenance therapy with ICIs is mostly not interrupted prior to RT. For management of RT associated pneumonitis under durvalumab the majority of 86.3% suggest corticosteroid therapy and 76.5% would postpone the next cycle of ICI therapy. The here obtained assessment and experiences by radiation oncologists reveal a large variability in practical handling of combined RT + ICI. Until scientific evidence is available a discussion for current clinical application of RT + ICI should be triggered. Interdisciplinary consensus guidelines with practical recommendations are required.

## Introduction

Immunotherapy (IT) and especially immune checkpoint blockade with PD1-(programmed cell death-1)/programmed cell death-ligand 1 (PD-L1) and CTLA-4 (cytotoxic T-lymphocyte-associated Protein 4) inhibitors have invaded the clinical routine and is revolutionizing the treatment of cancer. An increasing number of patients receive immune checkpoint inhibitors (ICIs) leading to an improved outcome in various cancer types and even metastatic diseases^1–4^. Nevertheless, there are important limitations, IT alone might not be sufficient to cure large tumor bulks, many patients are non-responders and long-term clinical benefits need to be studied. Moreover, depending on the molecular agent, and depending on the treatment regime (as standalone therapy or in combination with radiotherapy), side effects may be of concern. However, the new chance of IT to improve systemic disease control emphasizes the importance to optimize local tumor control, even in patients with metastatic diseases. Local tumor therapy can be performed by radiation therapy (RT) and the local treatment of all metastases can improve the overall survival under certain circumstances. Especially, stereotactic radiation therapy (SRT) is a commonly applied technique allowing safe escalation of radiation dose delivered in a very limited number of treatment fractions with high efficacy of local metastasis control. For a huge variety of malignancies^5–9^ and their oligometastatic disease^10–12^ SRT is commonly applied and achieves excellent local control rates with a tolerable toxicity^13–15^. Thus, it became evident that combining local RT (e.g., SRT) with systemic IT (e.g., ICIs) might be beneficial to further improve disease control in general. For instance, albeit it remains unclear if the risk of radiation necrosis might be elevated, a clear benefit in terms of outcome has been shown for IT and stereotactic treatment for brain metastases^16–18^. Therefore, in current clinical practice, many patients with advanced stage diseases (e.g., Melanoma, Non-small cell lung cancer) receive IT either simultaneously or sequentially to RT. Furthermore, there are many ongoing clinical trials evaluating the combination of RT with IT in different scenarios^19^.

On the one hand IT is emerging rapidly in clinical practice, also in combination with RT. On the other hand a lack of scientific evidence concerning toxicity and synergistic effects is reflected in missing guidelines and consequently in an uncertainty in clinical handling^20^. The current state of knowledge and evidence is predominantly based on preclinical studies. Most of the preclinical studies focus on efficacy of tumor therapies and strategies to improve tumor control rates but do not specifically investigate side effects of RT, IT or the combination of RT and IT. In order to address the open questions, clinical studies are required. Clinical scientific data on the safety of combined radioimmunotherapy are rare and rather reported a limited number of severe side effects^20–23^ depending on the therapy agent and irradiated region. Caution must be used for combination of SRT and targeted therapy to prevent severe toxicities for late reacting normal tissues such as vasculature when these are directly affected by targeted therapies such as antiangiogenic agents.

An overview of studies investigating side effects of RT + ICI was summarized by Hwang et al.^24^. Several studies focused either on sequential^25,26^ or also on concomitant ICIs^27,28^ with RT. Overall, grade ≥ 3 immune related adverse events (IRAEs) were found in 7% to 31% in mostly retrospective trials for combined RT + ICI^24^. Numerous studies report results on side effects of thoracic RT combined with ICI treatment^29–33^.

Treatment associated brain necrosis was investigated in multiple retrospective studies. Overall, study results are not as distinct as for lung toxicity. Between 20 and 30% occurrence of treatment associated brain necrosis were found overall^34–36^. Whereas some point out an increased rate of brain necrosis for combined radioimmunotherapy^21,37,38^, others cannot confirm these results^39^.

A variety of treatment schedules is used for fractionated and hypofractionated RT, thus potential effects of combined RT + ICI might vary. Furthermore, drug elimination half-life times of ICIs vary substantially^40^ (Table 1) and with them the effect causing intervals. Thus, also drug related toxicities depend on the treatment schedule. Also, there is a lack of scientific evidence based on large clinical trials for the handling of IRAEs for combined RT^41^. Therefore, an uncertainty in the practice of combination RT + ICI regarding therapy sequences, radiation fractionation regimes as well as potential pausing intervals of immunotherapy during RT for prevention of increased toxicity arises.

**Table 1:** Drug elimination half-life times t_1/2_ in days of checkpoint inhibitors^40^.

Generic name	t_1/2_ (days)
Ipilimumab	15
Atezolizumab	27
Avelumab	6.1
Durvalumab	21
Nivolumab	25
Pembrolizumab	27.3

Despite potentially enhanced side effects for RT + ICI, most current clinical trials suggest a combined therapy together with RT due to beneficial synergistic immunological effects^32,42–45^. For combination of pembrolizumab and RT for metastatic lung cancer a pooled analysis by Theelen et al. currently showed improved systemic tumor response rates of 19.7% with pembrolizumab alone versus 41.7% with RT concurrently or sequentially 1 week after the last dose of ICI therapy. A systematic overview of safety and potentials of RT + ICI was conducted by Jagodinsky et al. and Deutsch et al.^19,46^. However, the same immunologic mechanisms leading to an improved outcome could also cause more pronounced side effects.

The question of treatment scheduling is still open. Clinical evidence for combined RT + ICI from large randomized phase-III trials is missing. Thus, in this study we aim to assess the current clinical practice and experiences in application of combined RT + ICI by German-speaking radiation oncologists. We investigate radiation oncologists’ practical strategies and experience with side effects of RT + ICI and scheduling and compare them to the current state of evidence in the literature. By analyzing experts’ assessment, we try to provide a basis to open a discussion for management of clinical application of combined RT + ICI.

## Methods and materials

Experienced radiation oncologists developed a survey with 24 items on the practice of combined RT + ICI. The questionnaire focused on side effects of RT + ICI, common practice of scheduling, handling of adverse events and included characteristics of clinics and participants. Questions were designed to reveal whether there is a difference in application of RT + ICI for SRT or nfRT. Since this was an exploratory survey, the questions were specifically developed for this study. All questions were designed as multiple-choice questions and some allowing multiple answers. Some questions allowed for free responses in case of missing options. One question per page was displayed. Response to all questions was required to fulfill the questionnaire. Pilot testing was performed by volunteers who had experience in radiation oncology. Thereafter, we applied minor changes to enhance usability and readability. Consensus was defined for 75% agreement according to Diamond et al.^47^.

The questionnaire was implemented on the online platform “survio.com”. The platform ensured data protection and security (2048-bit SSL security, ISO/IEC 270001 standards, daily backups). The link was sent via e-mail to members of the German Society of Radiation Oncology (DEGRO). The participation was voluntary as well as anonymous. The survey was open for completion from September 1st 2020 until December 13th 2020. All statistical analysis was performed in a primarily descriptive way.

### Ethics approval and consent to participate

The department of radiation oncology of the Technical University of Munich has approved the study protocol. Survey participation was voluntary. Thereby participants gave their informed consent to take part in this study. All methods were performed in accordance with the relevant guidelines and regulations. No subjects under 18 years took contributed to this study.

## Results

### Participants characteristics

**Figure 1:**
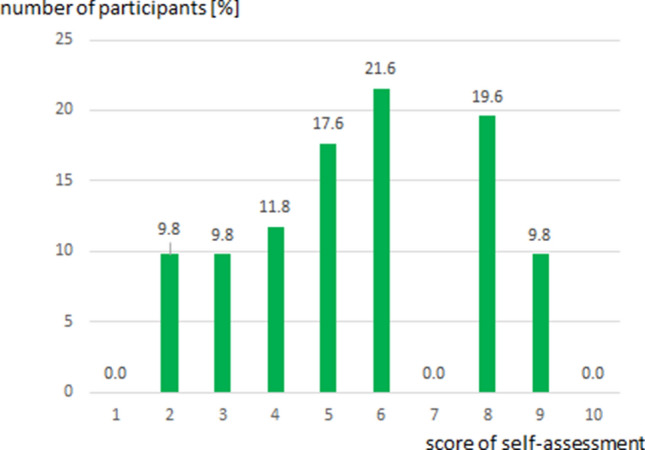
Self-assessment score in percent of the 51 participants. The scale reached from 1 to 10 where 1 refers to “very limited knowledge” and 10 refers to “excellent knowledge” on immune checkpoint inhibitors therapy.

In total, 51 participants of 1.291 invited completed the online questionnaire. Most of the participants (52.9%, 27/51) worked in a medical practice, 47.1% (24/51) worked in hospitals (25.5%, 13/51, in university hospitals). The majority of the participants (92.2%, 47/51) were specialists in radiation oncology. The rest were residents (7.8%, 4/51). An overview about the participants’ characteristics is depicted in Table 2. We also asked participants to rate their own knowledge about ICI therapy on a scale from 1 (very limited knowledge) to 10 (excellent knowledge). Data is depicted in Fig. 1. None of the participants felt to have very limited or excellent knowledge. The majority of 58.8% (30/51) estimated their own knowledge between a score of 5 to 8.

**Table 2:** Participant characteristics.

Characteristic	Absolute number	Percentage of all participants (%)
Practice	27	52.9
Hospital (non-university hospital)	11	21.6
University hospital	13	25.5
Specialists	47	92.2
Residents	4	7.8
Head of department	8	15.7
Head of private practice (and head of department)	9	17.6

### Observed side effects of combined RT + ICI

**Figure 2:**
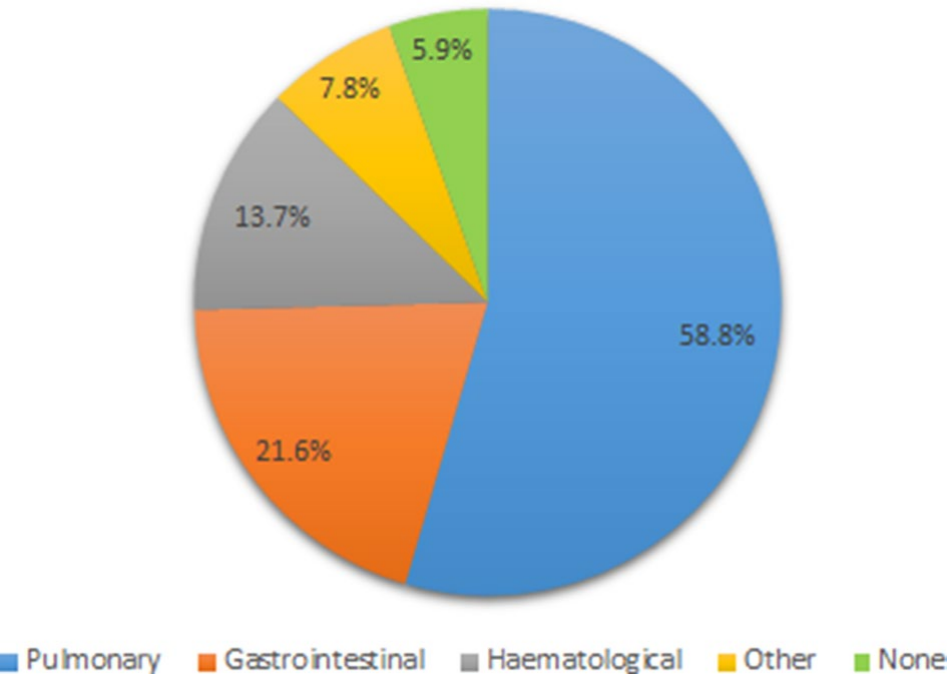
Toxicity, participants are most frequently concerned about under thoracic radioimmunotherapy (RT + ICI). Data is given in percent of all participants (N = 51). 4 (7.8%) out of 51 participants chose multiple answers. These are not depicted within the figure.

Results of the side effects that are noticed by participants are summarized in Table 3. Pulmonary toxicity was reported most frequently as a consequence of thoracic RT and IT with a PD1-/PD-L1-checkpoint inhibitor with almost 60%. Less frequently mentioned were gastrointestinal (21.6%, 11/51) and haematological (13.7%, 7/51) toxicities. Other side effects not explicitly given as a pre-defined answer were skin toxicity, fatigue and autoimmunological reactions (not specific to a certain organ type) in the questionnaire. Three participants also reported that they have not noticed any side effects of combined thoracic RT + ICI and one participant reported that this explicit combination RT + ICI is not performed at all. When we asked about the side effects that participants are concerned about more often under thoracic RT + ICI with PD1-/PD-L1 checkpoint inhibitors compared to RT without IT, pulmonary toxicity (58.8%, 30/51) still was most frequently mentioned. 31.4% (16/51) of the 51 participants of the questionnaire also saw haematological toxicity more frequently under combined RT + ICI than for RT alone. Gastrointestinal side effects are of pronounced concern according to 25.5% (13/51) of the participants rating. Two participants report that they noticed an increased toxicity of the thyroid. Other toxicities mentioned that are of more concern under RT + ICI refer to the liver, kidney and the pituitary gland. Seven participants reported that toxicity was not more frequently detected for a combined thoracic RT + ICI compared to RT or that they have not observed RT + ICI specific side effects at all. Data is visualized in Fig. 2.

**Table 3:** Toxicity observed for thoracic radioimmunotherapy with Immune Checkpoint Inhibitors (RT + ICI) and radiation therapy (RT) alone.

Toxicity	Most frequently observed under RT + ICI (thoracic) [number]/(%)	More frequently observed under RT + ICI (thoracic) compared to RT alone [number]/(%)
Pulmonary	30/58.8	30/58.8
Gastrointestinal	11/21.6	13/25.5
Haematological	7/13.7	16/31.4
Other	4/7.8	5/10.0
None	3/5.9	7/13.7
Multiple answers	4/7.8	14/27.5

Data is given in absolute number of answers and in percentage of the number of participants (N = 51).

We also asked for handling of potential side effects of RT + ICI. 19/51 (37.3%) have a standardized procedure with specific anamnesis of side effects. 27/51 (52.9%) regularly perform clinical examination before and during therapy. 33/51 (64.7%) participants perform oral education of potential side effects of RT + ICI and 31/51 (60.8%) use written forms for targeted education about RT + ICI.

### Pausing of ICI

**Figure 3:**
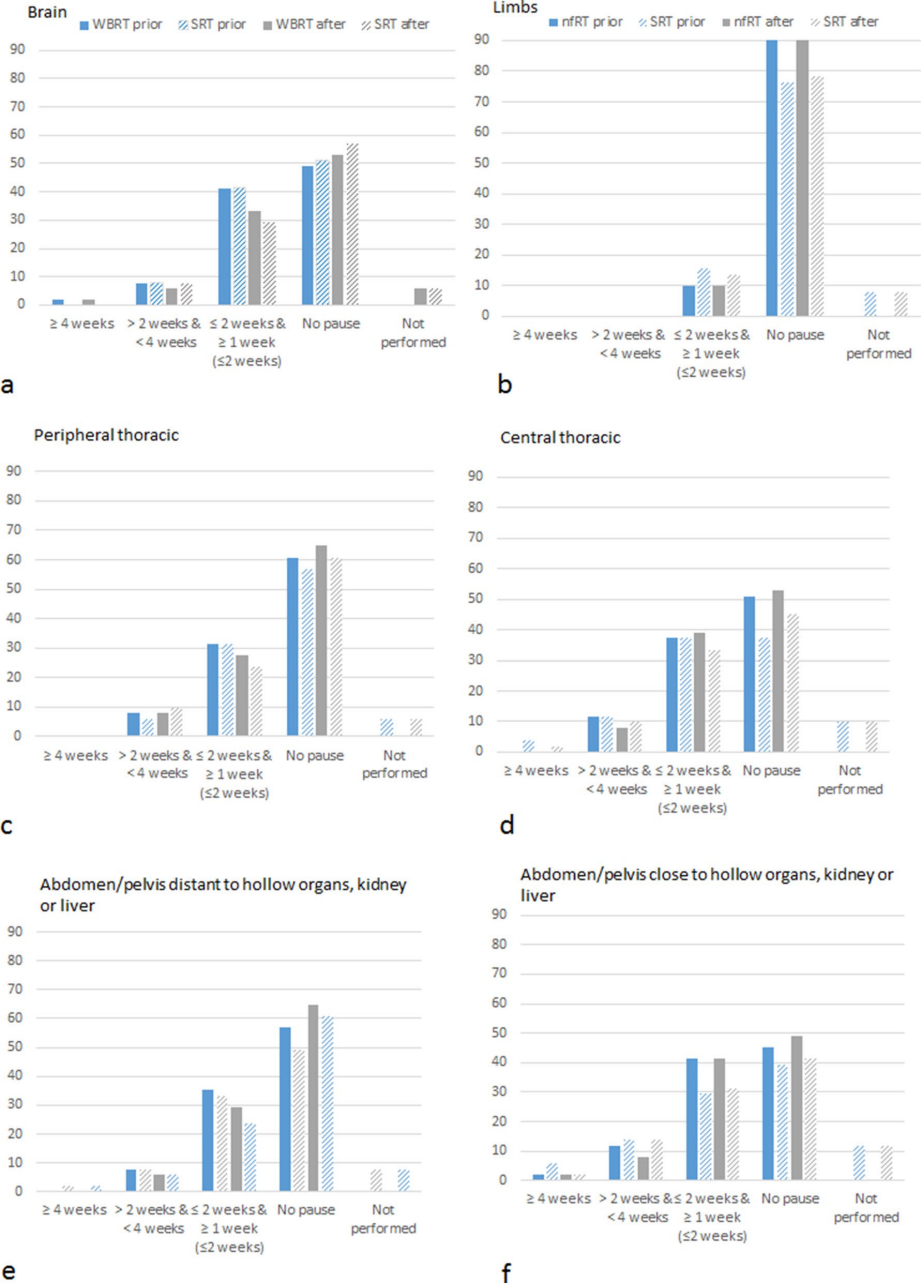
Handling of immune checkpoint inhibitors (ICI) treatment prior and after radiotherapy. Blue colored bars stand for “prior” radiotherapy, grey color stands for “after” radiotherapy. Full bars stand for normofractionated radiotherapy (nfRT) and dashed bars indicate stereotactic radiotherapy (SRT).

Fifteen questions dealt with the current clinical practice of pausing of ICI prior or after RT. We systematically asked whether participants pause ICI therapy during RT and the corresponding pausing interval prior and after radiation for various treatment locations. Each question differentiated between nfRT and hypofractionated SRT. Results are summarized in Supplement Table S1 and Fig. 3.

In 5.9% (3/51) (brain), 9.8% (5/51) (central thoracic region), 5.9% (3/51) (peripheral thoracic region), 11.8% (6/51) (abdominal/pelvic close to hollow organ, liver or kidney) and 7.8% (4/51) (abdominal/pelvic distant to hollow organ, liver or kidney) no SRT is performed.

#### Limbs

Even more clear is the handling during radiation of bone and soft tissue metastases of the limbs. Here, consensus was achieved. The vast majority of 90.2% (46/51) do not pause ICI therapy prior or after nfRT of metastases of the limbs. For SRT 76.5% (39/51) and 78.4% (40/51) do not pause ICI therapy prior or after radiation, respectively. 10% or even less of radiation oncologists pause ICIs between 1 and 2 weeks prior and after fractionated and stereotactic radiation of the limbs, respectively.

#### Brain

For brain treatment most of the participants do not pause ICI therapy during RT. Prior to whole brain irradiation 49% (25/51) do not pause ICI treatment and 51% (26/51) after, respectively. When SRT is performed 52.9% (27/51) do not pause ICIs prior and 56.9% (29/51) after RT. In case of ICI treatment interruption, most participants (41.2%) pause between 1 and 2 weeks prior to irradiation for whole brain radiation and less than 2 weeks after radiation. When SRT is performed 33.3% (17/51) suspend between 1 and 2 weeks prior to radiation and 29.4% (15/51) less than 2 weeks after. A minority of participants pause between 2 and 4 weeks prior and after brain radiation with almost no difference between the radiation techniques.

#### Thorax

For radiation treatment in the central thoracic region close to central bronchi, the heart or esophagus the majority do not suspend ICI prior (51.0% [26/51]) and after (52.9% [27/51]) nfRT or prior (37.3% [19/51]) and after (45.1% [23/51]) SRT. Compared to brain radiation more treating physicians (11.8% [6/51]) tend to pause ICIs longer (between 2 and 4 weeks) prior to radiation regardless of the fractionation schedule. Comparing pausing intervals for central and peripheral thoracic region a greater percentage of participants indicated to pause ICI treatment for central thoracic region (49% [25/51] for nfRT and 52.9% [27/51] for SRT) as for peripheral thoracic region (39.2% [20/51] for nfRT and 37.3% [19/51] for SRT).

#### Abdomen/pelvis

45.1% (23/51) do not suspend ICI therapy for RT of abdominal or pelvic tumors close to hollow organs. Almost the same number of participants which relates to 41.2% (21/51) pause IT between 1 till 2 weeks prior to nfRT. Results are similar for handling after fractionated radiation. 49.0% (25/51) do not suspend ICIs at all and 41.2% (21/51) pause for less than 2 weeks after RT. For SRT in the abdominal and pelvic regions 39.2% (20/51) and 41.2% (21/51) do not pause prior and after, respectively. 29.4% (15/51) and 31.4% (16/51) pause ICI treatment less than 2 weeks after SRT. Whereas for treatment in the thoracic region none of the participants pauses ICIs 4 weeks or longer prior or after radiation, single persons pause ICIs for abdominal and pelvic radiation for this period. 11.8% (6/51) and 13.7% (7/51) pause between 2 and 4 weeks prior and 7.8% (4/51) and 13.7% (7/51) after nfRT and SRT, respectively.

For radiation distant to hollow organs in the abdominal and pelvic region the trend goes to not pausing ICI therapy at all. 56.9% (29/51) and 64.7% (33/51) do not pause ICIs prior and after nfRT. 49.0% (25/51) and 60.8% (31/51) do not pause prior or after SRT, respectively. Also 7.8% (4/51) pause between 2 and 4 weeks prior to nfRT and SRT and 5.9% (3/51) pause between 2 and 4 weeks after.

#### Maintenance treatment with durvalumab for NSCLC

Results for RT handling during ICI maintenance treatment are presented in Table 4. 64.7% (33/51) of all participants start durvalumab maintenance therapy between 1 and 2 weeks after RT. 21.6% (11/51) wait more than 2 weeks and 11.8% (6/51) start within less than a week. For SRT during maintenance therapy over 68.8% (35/51) do not suspend ICIs prior to nfRT and more than 56% (29/51) for SRT, respectively. After radiation less pause ICI maintenance treatment. The longest interval of ICI treatment interruption, that is applied, was between 2 and 4 weeks (5.9% [3/51] for nfRT and 7.8% [4/51] for SRT).

**Table 4:** Practical handling of durvalumab maintenance therapy after chemoradiotherapy (CRT) for non small cell lung cancer (NSCLC) and pausing times prior and after radiotherapy (RT) of metastases during maintenance therapy with programmed cell death-ligand 1 (PD-L1).

	**Time after CRT before start of durvalumab maintenance treatment**									
	>2 w	≤ 2 w	≤ 1 w	Other						
	11 (21.6%)	33 (64.7%)	6 (11.8%)	1 (2%)						

RT technique of metastasis	PD-L1 ICI pausing interval prior to RT					PD-L1 ICI pausing interval after to RT				
	≥ 4 w	> 2w and < 4w	≤ 2w& ≥ 1w	No pause	Not perf	> 4 w	> 2w and ≤ 4w	≤ 2 w	No pause	Not perf
**RT during PD-L1 ICI maintenance treatment**										
fRT	0	3 (5.9%)	13 (25.5%)	35 (68.6%)	0	0	3 (5.9%)	11 (21.6%)	37 (72.5%)	0
SRT	0	3 (5.9%)	16 (31.4%)	29 (56.9%)	3 (5.9%)	0	4 (7.8%)	13 (25.5%)	31 (60.8%)	3 (5.9%)

Data is provided in absolute numbers of answers and in percentage of the total number of participants (N = 51). “Not perf.” indicates that this type of therapy is not performed. “w” stands for weeks.

*nfRT* normofractionated radiotherapy, *SRT* stereotactic radiotherapy.

### Adverse event handling and steroid therapy

One question addressed the issue of IRAEs under ICI treatment. The question focused on the practical handling of RT within the organ affected by the IRAE and distant from it. The results are summarized in Table 5. 47.1% (24/51) would instantly begin RT distant from the organ affected by the IRAE. 74.5% (38/51) would start radiation within the same organ that was affected by IRAE after completely resolved IRAE. A small number of participants would not perform radiation at all.

**Table 5:** Practical handling and pausing intervals after an immune related adverse event (IRAE) until the start of radiotherapy (RT) depending of the location of RT (N = 51).

RT location	Pausing interval after IRAE to start of RT			
	After healed IRAE	During IRAE	After incompletely healed IRAE in patient history	Never
RT of organ affected by IRAE	38 (74.5%)	0	10 (19.6%)	3 (5.9%)
RT distant organ affected by IRAE	16 (31.4%)	24 (47.1%)	9 (17.6%)	2 (3.9%)

We also asked for handling of prophylactic corticosteroid therapy for intracranial SRT during ICI treatment for prevention of brain edema. 9/51 (17.6%) participants would administer corticosteroid therapy. The same number of participants would not. 33/51 (64.7%) participants would only give prophylactic corticosteroids in case of large or critically located brain metastases.

In case of RT associated pneumonitis under maintenance therapy with durvalumab 44/51 (86.3%) participants would give corticosteroid therapy and 39/51 (76.5%) would postpone the next cycle of durvalumab. None of the participants explicitly decided not to give corticosteroid therapy.

## Discussion

We conducted a survey on the current clinical practice and experiences in application of combined RT + ICI by German-speaking radiation oncologists. The questionnaire revealed a distinct uncertainty and diversity in clinical handling of combined RT + ICI. Obviously, there is the feeling of lacking knowledge concerning ICI treatment in the radiation oncology community in the German speaking countries. Although, the vast majority of participants were specialists (47) in radiation oncology only 15 assessed their status of knowledge greater than seven on a scale from 1 (low) to ten (excellent).

When we asked radiation oncologists for their assessment regarding side effects under combined thoracic RT and ICI treatment, pulmonary toxicity seems to be most frequently expected. Obviously, due to the approval of the applied drugs, a majority of studies deal with NSCLC. Thus, thoracic RT might be a common intervention and due to the participants’ evaluation, pulmonary toxicity can be emphasized.

For cranial RT approximately half of the participants do not pause ICI therapy at all prior or after RT. 41.2% and 29.4% start ICI treatment in less than 2 weeks after whole brain RT and SRT, respectively. Scientific clinical data on the central nervous toxicity is mainly based on retrospective studies analyzing increased treatment associated necrosis, mostly radiographically diagnosed^35,37,38^, other studies do not emphasize this trend^38,39^. The results obtained by this questionnaire reveal that radiation oncologists suggest to either not pause or shortly pause ICI therapy during brain irradiation. There is a trend for pausing for whole brain irradiation rather than for intracranial SRT. This trend in clinical practice cannot be directly related to the above mentioned scientific findings. There is currently no evidence for short term ICI interruption prior or after intracranial brain radiotherapy with respect to toxicity. However, results obtained here, suggest that some clinicians probably feel more safe by pausing around radiotherapy.

Regarding thoracic RT, we distinguished between central and peripheral radiation. Approximately half of the participants (less than 52.9%) do not pause ICIs for central thoracic nfRT and less than 45.1% for SRT. For peripheral thoracic lesion even more do not suspend ICI treatment for nfRT and SRT, respectively. In case of pausing there is a clear trend towards a short interruption for 1 to 2 weeks around RT, even more pronounced for nfRT than for SRT. A very limited number of participants suggest an ICI interruption even for 4 weeks or more around central thoracic RT. In the literature some studies show no increase in higher grade pneumonitis caused by combined ICIs with RT^29,30^, however, an increased rate of pneumonitis including all grades was observed. In the PEMBRO-RT trial studying ICI therapy with pembrolizumab after thoracic SRT pneumonia rates were higher in the RT + ICI arm compared to pembrolizumab monotherapy (26% vs. 8%). The pneumonitis rate in the RT + ICI arm was 11%. Similar results were found by Bauml et al.^31^ who studied NSCLC patients receiving pembrolizumab after SRT.

In the phase-III PACIFIC trial^33^ studying consolidation therapy with durvalumab after CRT, an increased rate of all grade pneumonitis was observed in the durvalumab group without an increase of higher grade pneumonitis. Currently, a large number of clinical trials with variable RT fractionation and RT + ICI treatment regimens are ongoing^24^. In this survey, approximately half of the participants do not pause ICIs for thoracic RT. However, participants distinguish here between central and peripheral thoracic RT. To our knowledge, there is currently no scientific evidence available on differences in side effects with respect to tumor location. Moreover, a trend to make a short ICI treatment interruption for application of high local doses by SRT was found. Together with the reported side effects collected here, participants are concerned of a potentially increased pulmonary side effect profile as reported in some clinical trials mentioned above. However, our study results show a diverse behavior in clinical practice. This reveals the uncertainty based on the lacking scientific evidence in management on combined ICI therapy during thoracic RT. We believe, the professional society longs for scientific evidence and resulting distinct guidelines.

Interestingly, when it comes to abdominal or pelvic RT close to critical organs (hollow organs, kidney, liver) more radiation oncologists suggest pausing ICI treatment. 41.2% and 29.4% pause ICI treatment 1 to 2 weeks prior to fractionated RT and SRT, respectively. Here scientific evidence for the optimal treatment schedule is lacking. The potential effect of 1 to 2 weeks pausing around abdominal RT with regard to side effects can be questioned. We suppose that the current clinical practice revealed by this questionnaire traced back to the side effect profile of ICIs and abdominal IRAEs such as hepatitis and other gastrointestinal toxicity. Clinicians might be afraid of causing severe side effects, hence most likely a feeling of safety is transported by suspending immunotherapy around RT.

Consensus was observed for RT of bone and soft tissue metastases during ICI therapy. 90.2% do not pause ICI therapy around nfRT. And 76.5% and 78.4% do not pause prior to SRT and after, respectively. No one pauses longer than 2 weeks around nfRT or SRT. These results may be explained by the predominantly palliative character of RT to bone and soft tissue metastases combined with the expectation of no pronounced side effects due to combined RT + ICI. Collectively, the professional community seems to tend towards no interruption for bone and soft tissue RT.

In summary, for evaluation of the optimal schedule for combined ICI therapy with RT, side effects should be considered as well as synergistic effects. The current perspective is based on the hypothesis of induction of antitumor immune response by RT enhancing the effect of ICI treatment distant to the treated region. Several studies focusing on combined RT + ICI are currently ongoing. A systematic overview can be found in the literature^24,46^. The underlying mechanisms include the release of antigens from tumor cells and upregulation of immunomodulatory cell surface molecules triggering antitumor immune response^48,49^. RT also induces the release of free radicals and cortisol having an immunosuppressive effect that typically reverts after 7–15 days. PD-L1, activated by tumor cells, is upregulated in response to RT resulting in a synergistic effect or RT and ICIs. The optimal treatment sequence should therefore be RT followed by ICI therapy with a delay of 1 to 2 weeks proposed by Mielgo-Rubio et al.^50^.

The only published randomized phase-III trial for durvalumab after CRT for stage III NSCLC is the PACIFIC trial^33^. Due to the superior clinical results obtained and its relevance in current clinical routine, we also asked participants of the questionnaire when they usually start administering durvalumab after CRT. 64.7% start in between 2 weeks after CRT. 21.6% wait longer (more than 2 weeks) and 11.8% wait less than 1 week after CRT. The study protocol suggested administration of durvalumab one to 42 days after RCT. A recent post-hoc analysis of the PACIFIC data by Faivre-Finn et al. suggests no difference in starting adjuvant durvalumab ≥ 14 days or before^51^. The here collected data shows that all of the participants administer durvalumab in accordance with the PACIFIC study protocol.

Importantly, scheduling RT during maintenance therapy with ICIs might be an important issue to address. When we asked for ICI interruption during maintenance therapy for RT, the majority of participants of 68.6% and 56.9% do not pause ICI therapy prior to nfRT or SRT, respectively. Interestingly, even more than 72.5% and 60.8% do not pause after nfRT and SRT. In case of interruption of consolidation therapy, there is a trend of pausing less than 2 weeks around ICI therapy for SRT in accordance with the usual drug administration every 2 or 3 weeks. Here, again data from large randomized trials is missing. A number of currently ongoing study focuses on concurrent RT + ICI (NCT03833154, NCT03519971, NCT02434081, NCT03706690). An overview of ongoing studies can be found in the articles of Botticella et al. and Ko et al.^52,53^.

In case of IRAE during ICI therapy 74.5% would start elective RT after complete resolving in case RT includes the organ affected by the IRAE. In case RT does not include the organ affected by the IRAE 31.4% would wait with the beginning of RT after complete healing. 47.1% would not wait at all. To our knowledge there is no distinct evidence for correct oncological management in this situation. This uncertainty is reflected by the distribution of answers. However, by 74.5% almost consensus (defined by 75%) was reached to wait with elective RT including an organ affected by IRAE after complete resolving.

An important question arising addresses the handling of ICI therapy and concurrent corticosteroid therapy. 64.7% of all participants suggest administering prophylactic corticosteroid for prevention of edema prior or concurrent to cranial SRT only in case of a large or critically located lesion. Consensus was achieved for management of radiation pneumonitis requiring therapy during durvalumab treatment of NSCLC. 86.3% would give corticosteroids and 76.5% would postpone the next cycle of ICI therapy. Although guidelines exists for management of IRAEs for patients treated with ICIs^54^, guidelines for the handling of side effects in combination with radiotherapy are lacking^41^. Mielgo-Ruibio et al.^50^ suggest that re-challenging with durvalumab should be considered after resolution or downgrading to grade 1. We think, the here collected evaluation of radiation oncologists shows quite a distinct tendency for administration of corticosteroids if necessary and pausing or postponing of maintenance therapy.

Clearly, the data obtained here, rather aims to assess the current clinical practice and strategies and cannot provide statistically significant data on side effects of RT + ICI. Moreover, the number of participants was small, thus results might not be representative for the entire group of radiation oncologists. Caution should be warranted when interpreting the here presented results, since they represent rather clinicians’ individual experiences and opinions and cannot serve as a substitute for scientifically gained data. For this, large clinical trials are required.

## Conclusion

With this survey, we demonstrate current clinical practice regarding scheduling and management of side effects of combined RT + ICI. The survey revealed a variability in practical clinical handling of combined RT + ICI reflecting the current lack of scientific evidence. Particular spreading of answers was noticed for management of interruption for RT during ICI treatment. Radiation oncologists here rather suggest not to pause ICI treatment for RT with a slight difference between nfRT and SRT. For SRT rather a short interruption of 1 to 2 weeks around ICI treatment seems to be practically applied. Pausing of ICI therapy prior to RT was generally more evident than after RT. Clinicians seem to be concerned about an increased risk of pulmonary and gastrointestinal toxicities and cautiously apply concurrent RT + ICI in the corresponding organs. Consensus was reached for no interruption of ICIs during bone irradiation. For the management of side effects during ICI maintenance therapy also a consensus favoring treatment of IRAEs and pausing ICI therapy was achieved.

Summarizing, the here obtained assessment and experiences by radiation oncologists of the DEGRO might open a discussion for current clinical application of combined RT + ICI and reveal the necessity of currently missing guidelines.

## Supplementary Information

Supplementary Information.
