# Supplementary material for: Evaluation of practical experiences of German speaking radiation oncologists in combining radiation therapy with checkpoint blockade
Source: Sci Rep. 2021 Apr 7;11:7624. doi: 10.1038/s41598-021-86863-2 (PMC8027172; doi:10.1038/s41598-021-86863-2)
Supplement: Supplementary file 1 — Supplementary Information. [file 41598_2021_86863_MOESM1_ESM.docx]

Evaluation of practical experiences of German speaking radiation oncologists in combining radiation therapy and checkpoint blockade

Kim M. Kraus^1*^, Julius C. Fischer^1^, Kai J. Borm^1^, Marco M. E. Vogel^1^, Steffi. U. Pigorsch^1, 3^, Michal Devečka^1^, Stephanie E. Combs^1,2,3^

^1^*Department of Radiation Oncology, School of Medicine, Technical University of Munich (TUM), Klinikum rechts der Isar, Munich, Germany.*

*^2^Helmholtz Zentrum München (HMGU), Institute of Radiation Medicine (IRM), Department of Radiation Sciences (DRS), Neuherberg, Munich, Germany.*

*^3^Deutsches Konsortium für Translationale Krebsforschung (DKTK), Partner Site Munich, Munich, Germany.*

*Corresponding author: ^*^kimmelanie.kraus@mri.tum.de, phone: 0049 89 4140 5373*

Supplement Table S1: Pausing intervals of ICI prior and after RT for normofractionated radiotherapy (nfRT) and stereotactic radiotherapy (SRT). Data are provided in absolute numbers of answers and in percentage of the total number of participants (N=51). WBRT stands for whole brain radiotherapy.

| Treatment location | RT | Prior to RT | | | | | After RT | | | | |
| --- | --- | --- | --- | --- | --- | --- | --- | --- | --- | --- | --- |
|  |  | ≥ 4 w | >2w &< 4w | ≤2w&≥1w | No pause | Not perf. | > 4 w | >2w&≤4w | <= 2 w | No pause | Not perf. |
| Brain | WBRT | 1  (2.0%) | 4  (7.8%) | 21  (41.2%) | 25  (49.0%) | 0 | 0 | 4  (7.8%) | 21  (41.2%) | 26  (51.0%) | 0 |
|  | SRT | 1  (2.0%) | 3  (5.9%) | 17  (33.3%) | 27  (52.9%) | 3  (5.9%) | 0 | 4  (7.8%) | 15 (29.4%) | 29  (56.9%) | 3  (5.9%) |
| Central thoracic region | nfRT | 0 | 6  (11.8%) | 19  (37.3%) | 26  (51.0%) | 0 | 0 | 4  (7.8%) | 20 (39.2%) | 27  (52.9%) | 0 |
|  | SRT | 2  (3.9%) | 6  (11.8%) | 19  (37.3%) | 19  (37.3%) | 5  (9.8%) | 1 (2.0%) | 5  (9.8%) | 17 (33.3%) | 23  (45.1%) | 5  (9.8%) |
| Peripheral thoracic region | nfRT | 0 | 4  (7.8%) | 16  (31.4%) | 31  (60.8%) | 0 | 0 | 4 (7.8%) | 14 (27.5%) | 33  (64.7%) | 0 |
|  | SRT | 0 | 3  (5.9%) | 16  (31.4%) | 29  (56.9%) | 3  (5.9%) | 0 | 5 (9.8%) | 12 (23.5%) | 31  (60.8%) | 3  (5.9%) |
| Abdomen/pelvis (close to hollow organs, liver or kidney) | nfRT | 1  (2.0%) | 6  (11.8%) | 21  (41.2%) | 23  (45.1%) | 0 | 1  (2.0%) | 4 (7.8%) | 21 (41.2%) | 25  (49.0%) | 0 |
|  | SRT | 3  (5.9%) | 7  (13.7%) | 15  (29.4%) | 20  (39.2%) | 6 (11.8%) | 1  (2.0%) | 7 (13.7%) | 16 (31.4%) | 21  (41.2%) | 6  (11.8%) |
| Abdomen/pelvis (distant to hollow organs, liver or kidney) | nfRT | 0 | 4  (7.8%) | 18  (35.3%) | 29  (56.9%) | 0 | 0 | 3  (5.9%) | 15 (29.4%) | 33  (64.7%) | 0 |
|  | SRT | 1  (2.0%) | 4  (7.8%) | 17  (33.3%) | 25  (49.0%) | 4  (7.8%) | 1  (2.0%) | 3  (5.9%) | 12 (23.5%) | 31  (60.8%) | 4  (7.8%) |
| Limbs | nfRT | 0 | 0 | 5  (9.8%) | 46  (90.2%) | 0 | 0 | 0 | 5 (9.8%) | 46  (90.2%) | 0 |
|  | SRT | 0 | 0 | 8  (15.7%) | 39  (76.5%) | 4  (7.8%) | 0 | 0 | 7 (13.7%) | 40  (78.4%) | 4  (7.8%) |
